# Transcription factor CitERF71 activates the terpene synthase gene *CitTPS16* involved in the synthesis of *E*-geraniol in sweet orange fruit

**DOI:** 10.1093/jxb/erx316

**Published:** 2017-09-07

**Authors:** Xiang Li, Yaying Xu, Shuling Shen, Xueren Yin, Harry Klee, Bo Zhang, Kunsong Chen

**Affiliations:** 1 Laboratory of Fruit Quality Biology/Zhejiang Provincial Key Laboratory of Horticultural Plant Integrative Biology, Zhejiang University, Zijingang Campus, Hangzhou, PR China; 2 Horticultural Sciences, Plant Innovation Center, Genetics Institute, University of Florida, Gainesville, FL, USA

**Keywords:** AP2/ERF, fruit, ripening, terpene synthase, transcription factor, volatile

## Abstract

The unique flavor of *Citrus* fruit depends on complex combinations of soluble sugars, organic acids, and volatile compounds. The monoterpene *E*-geraniol is an important volatile, contributing to flavor in sweet orange (*Citrus sinensis* Osbeck). Moreover, antifungal activity of *E*-geraniol has also been observed. However, the terpene synthase (TPS) responsible for its synthesis has not been identified in sweet orange. Terpene synthase 16 (CitTPS16) was shown to catalyze synthesis of *E*-geraniol *in vitro*, and transient overexpression of *CitTPS16* in fruits and leaves of Newhall sweet orange resulted in *E*-geraniol accumulation *in vivo*. Having identified the responsible enzyme, we next examined transcriptional regulation of *CitTPS16* in the fruit. Among cloned members of the AP2/ERF transcription factor gene family, *CitERF71* showed a similar expression pattern to *CitTPS16*. Moreover, CitERF71 was able to activate the *CitTPS16* promoter based on results from transient dual-luciferase assays and yeast one-hybrid assays. EMSAs showed that CitERF71 directly binds to ACCCGCC and GGCGGG motifs in the *CitTPS16* promoter. These results indicate an important role for CitERF71 in transcriptional regulation of *CitTP16* and, therefore, in controlling production of *E*-geraniol in *Citrus* fruit.

## Teaser

Synthesis of the sweet orange fruit volatile *E*-geraniol is catalyzed by CitTPS16, and the *CitTPS16* gene is transcriptionally regulated during ripening

## Introduction

Plants produce a wide variety of volatile organic compounds (VOCs) during growth and development, mediating many aspects of responses to the environment (Chen and Viljoen, 2010). For fruits, volatiles are important as attractants for seed-dispersing organisms and are linked to flavor and nutritional quality (Goff and Klee, 2006). As economically important fruits, *Citrus* are rich in volatile terpenoids. Among these compounds, monoterpenes are major components of flavor-related volatiles (Lan-Phi *et al.*, 2009).

*E*-geraniol is an acyclic monoterpene alcohol released from many plants, including roses and herbs (Charles and Simon, 1992; Antonelli *et al.*, 1997; Rao *et al.*, 2000; Iijima *et al.*, 2004), and is used in cosmetics and flavor industries due to its pleasant rose-like smell. GC–olfactometry demonstrated that *E*-geraniol is a characteristic flavor component of daidai (*Citrus aurantium*) peel oil (Song *et al.*, 2000). Synthesis of many volatiles is related to defense in plants. For example, *E*-geraniol has been reported to function as an antibacterial compound against *Xcc* (*Xanthomonas citri* subsp. *citri*) that causes bacterial canker (Mizaei-Najafgholi *et al.*, 2017). Antifungal activity of *E*-geraniol was also observed in *Citrus* (Singh *et al.*, 2010; Hasija *et al.*, 2015; Sharma *et al.*, 2016; Geraci *et al.*, 2017). In rough lemon, vapor treatment with *E*-geraniol induces expression of defense-related genes such as *RlemLOX* (Yamasaki and Akimitsu, 2007). Thus, beyond its contribution to flavor, *E*-geraniol might function as a signal molecule to activate defense signaling pathways against fungal attack (Shishido *et al.*, 2012).

Plant terpenoids are derived from either the cytosolic mevalonic acid (MVA) pathway or the plastidic methylerythritol phosphate (MEP) pathway (Rodríguez-Concepción and Boronat, 2002), synthesized from the two C5-isoprene building units, isopentenyl diphosphate (IPP) and dimethylallyl diphosphate (DMAPP). Prenyltranferase geranyl diphosphate synthase catalyzes condensation of IPP and DMAPP to produce the immediate precursor of monoterpenes [geranyl diphosphate (GPP)]. After the formation of these precursors, most of the monoterpenes are synthesized by downstream terpene synthases (TPSs; Trapp and Croteau, 2001). Recently, a TPS-independent pathway for monoterpene biosynthesis was identified in rose. Involvement of a nudix hydrolase, RhNUDX1, in the formation of rose geraniol was observed as an non-canonical pathway (reviewed in Sun *et al.*, 2016). For *Citrus* fruit, monoterpene synthase genes that are associated with ocimene and limonene have been characterized (Shimada *et al.*, 2005; Rodríguez *et al.*, 2011), implying that monoterpenes seem to be synthesized by canonical pathways in the genus *Citrus*. Although it is an important volatile component in *Citrus* fruit, the TPS enzyme responsible for *E*-geraniol biosynthesis in sweet orange has not yet been characterized.

Transcriptional regulation of terpenoids has been observed in plants. In *Arabidopsis thaliana*, *AtMYC2* regulates synthesis of sesquiterpenes by binding to the promoter of *AtTPS11* and *AtTPS21* (Hong *et al.*, 2012). In cotton, a WRKY-binding element (W-box) is present in the promoter of the (+)-δ-cadinene synthase-A gene *CAD1-A* (Xu *et al.*, 2004). The APETALA2/ethylene-response factor (AP2/ERF) superfamily contains four subfamilies: DREB, ERF, AP2, and RAV (Sakuma *et al.*, 2002). Involvement of AP2/ERFs in transcriptional regulation of terpenoids has been described for *Artemisia annua AaERF1* and *AaERF2* (Yu *et al.*, 2012), and *Zea mays ZmEREB58* (Li *et al.*, 2015). However, in fruits, AP2/ERF transcription factors that interact with TPS promoters, activate TPS expression, and thus contribute to terpenoid formation have not been identified.

In the present study, using the *C. sinensis* genome database, and based on sequence homology and phylogenetic analysis, TPS genes with potential functions in production of volatile monoterpenes were screened. Both *in vitro* assay of recombinant protein and *in vivo* transient expression in Newhall sweet orange (*C. sinensis* Osbeck) indicated that *CitTPS16* is responsible for *E*-geraniol biosynthesis. Furthermore, CitERF71 could directly bind to and activate the *CitTPS16* promoter. The action on the promoter of the target gene *CitTPS16* was studied using tobacco dual-luciferase assays yeast one-hybrid (Y1H) analysis, and EMSAs.

## Materials and methods

### Plant materials

Fruits of Newhall sweet orange (*C. sinensis* Osbeck) were obtained from a commercial orchard in Songyang (Zhejiang, China) at six different developmental stages, 45, 75, 105, 135, 165, and 195 d after full bloom (DAFB). Fruits of hybrid Huyou (*Citrus paradisi*), and mandarin Ponkan (*Citrus reticulata* Blanco) were obtained from a commercial orchard in Quzhou (Zhejiang, China) and collected at the mature stage, 205 DAFB and 225 DAFB, respectively. The fruit were transported to the lab, and peels were sampled, frozen in liquid nitrogen, and then stored at –80 °C until use.

### Volatile *E*-geraniol analysis by GC-MS

Volatile analysis was carried out according to our previous study (Shen *et al.*, 2016). Frozen tissues were ground in liquid nitrogen and transferred into a vial containing saturated sodium chloride solution. After vigorous vortexing, the samples were incubated at 40 °C for 30 min with continuous agitation (600 rpm). Following this, an SPME fiber coated with 50/30 μm divinylbenzene/carboxen/polydimethylsiloxane (DVB/CAR/PDMS) (Supelco Co., Bellefonte, PA, USA) was used to extract the volatiles. *E*-geraniol detection was analyzed using an Agilent 7890A gas chromatograph coupled to an Agilent 5975C Network Mass Selective Detector (MS, insert XL MSD with triple-axis detector). Volatiles were separated using a HP-5MS column (30 m×0.25 mm×0.25 μm; J&W Scientific, Folsom, CA, USA). Helium was used as a carrier gas at 1.0 ml min^–1^. The oven temperature was programmed to start at 40 °C for 3 min, and then ramped to 70 °C at a rate of 3 °C min^–1^, followed by a second ramp to 130 °C at a rate of 1 °C min^–1^, and a third ramp to 230 °C at a rate of 15 °C min^–1^. MS conditions were as follows: ion source, 230 °C; electron energy, 70 eV; GC-MS interface zone, 250 °C, and a scan range of 35–350 mass unit. Internal standards were used for compensating for differences between samples. To identify *E*-geraniol, the retention time was compared with that of an authentic standard purchased from Sigma-Aldrich. Identification of *E*-geraniol was further validated by comparing its electron ionization mass spectrum and Kovats retention index (RI) with those in the NIST/EPA/NIH Mass Spectral Library and Wiley Registry of Mass Spectral Data. Kovats RI was calculated by injecting C7–C21 linear alkane mixture standards.

### Gene isolation and sequence analysis

Total RNA was extracted from frozen tissues according to Zhang *et al.* (2006). The total RNA obtained was treated with TURBO DNase (Ambion) for cDNA synthesis using an iScript cDNA Synthesis Kit (Bio-Rad). Full-length coding sequences (CDSs) of *CitAP2/ERF* and *CitTPS16* were cloned using primers described in Supplementary Table S1 at *JXB* online, using the *C. sinensis* genome database (http://citrus.hzau.edu.cn/) as a reference. For the promoter of *CitTPS16*, an ~2500 bp sequence was cloned and sequenced. The promoter sequence was confirmed by comparing it with the genome database. Alignment of sequences was carried out with the ClustalX program, and a phylogenetic tree was generated with FigTree v1.4.2.

### Recombinant protein of CitTPS16 and enzymatic activity assay

For functional studies, *CitTPS16* was subcloned in truncated form in order to exclude the putative plastid-targeting signal from being expressed, because this can lead to the formation of inclusion bodies (Williams *et al.*, 1998). The clone was truncated and religated in the pET6×HN vector (Clontech, Palo Alto, CA, USA). Primers and vectors used are listed in Supplementary Table S1. The recombinant N-terminal His-tagged protein was expressed by autoinduction in *Escherichia coli* BL21 (DE3) pLysS (Promega, Madison, WI, USA). To express the recombinant protein, 10 ml of the overnight culture were combined with 500 ml of an LB liquid medium with 100 μg ml^–1^ ampicillin. Isopropyl-β-d-galactopyranoside (IPTG) was added to a final concentration of 1 mM to induce the production of recombinant protein. After incubation at 16 °C at 150 rpm for 18 h, the cells were harvested by centrifugation (4000 *g*, 4 °C, 15 min) and resuspended in 1× phosphate-buffered saline (PBS; 1.37 M NaCl, 26.8 mM KCl, 20.3 mM Na_2_HPO_4_, and 17.6 mM KH_2_PO_4_, pH 7.2). The cells were disrupted by sonication, and the supernatant was purified using a TALON Spin column (Clontech) following the manufacturer’s instruction. SDS–PAGE was carried out using Tris–HCl buffer (pH 7.5) and the protein was visualized by Coomassie brilliant blue. A 30 μg aliquot of purified recombinant CitTPS16 and GPP or farnesyl diphosphate (FPP) (100 μM final concentration: Sigma-Aldrich, Saint Louis, MO, USA) was put into a 4 ml headspace vial containing 900 μl of buffer with Mn^2+^ and Mg^2+^ as cofactors. Before capping of the vial, 5 μl of 1-hexanol (10%, v/v) was added as an internal standard and incubated at 30 °C (room temperature) for 2 h. Then the samples were incubated at 40 °C for 20 min with continuous agitation (600 rpm). Volatile analysis was carried out as described for *E*-geraniol analysis mentioned above. At least three biological replicates were performed for enzymatic assays.

### Transient overexpression in Newhall sweet orange fruits and leaves

Transient expression analysis was performed on Newhall sweet orange fruit peels and leaves on trees, which are planted in a greenhouse (28 ± 2 °C in the day time, 25 ± 2 °C at night). The empty vector (SK) and constructs containing *CitTPS16* (target gene) were carried by *Agrobacterium* cultures and then infiltrated into the same fruit on opposite sides of the equatorial portion (Yin *et al.*, 2016), and the same leaf on the left and right side separated by the midrib. After infiltration, the Newhall sweet orange fruit peels and plants were grown for 5 d and 7 d, respectively. The peel near (<6 mm) and the leaf near (<5 mm) the infiltration point (without including the infiltration) were collected and immediately frozen in liquid nitrogen. These samples were stored at –80 °C for volatile analysis (1 g of fruit peel and 0.1 g of leaves for measurements for each replicate). There were three biological replicates for transient overexpression analysis. The volatiles were analyzed using GC-MS as mentioned above. The primers are shown in Supplementary Table S1.

### Real-time quantitative PCR

The oligonucleotide primers of *CitAP2/ERF* genes used for real-time quantitative PCR were designed by Xie *et al.* (2014). The *CitTPS16* and *CitERF71* primers were designed by primer3 (http://frodo.wi.mit.edu/primer3) and are described in Supplementary Table S1. The specificity of the primers was tested with melting curves and sequencing of PCR products. Real-time PCR was carried out with a Ssofast Eva Green Suppmix Kit using a CFX96 instrument (Bio-Rad). The PCR program was initiated with a preliminary step of 5 min at 95 °C, followed by 50 cycles at 95 °C for 10 s, 60 °C for 10 s, and 72 °C for 15 s. Melting curve analyses were performed for each gene, at the end of each run. Abundance of cDNA templates was monitored with citrus β-actin (Pillitteri *et al.*, 2004). ∆∆Ct was used to calculate the relative expression level of genes (Livak and Schmittigen, 2001).

### Dual-luciferase assay

Transactivation activities of AP2/ERF on the target promoter were measured with dual-luciferase assays as previously reported (Yin *et al.*, 2010; Xu *et al.*, 2014). Full-length *CitERF71* sequences were amplified with the primers described in Supplementary Table S1 and were inserted into a pGreenII 0029 62-SK vector. The promoter of *CitTPS16* (2437 bp) was constructed in the pGreenII 0800-LUC vector. All constructs were individually electroporated into *Agrobacterium* GV3101 and stored as glycerol stocks at –80 °C. *Agrobacterium* cultures were prepared with infiltration buffer (10 mM MES, 10 mM MgCl_2_, 150 mM acetosyringone, pH 5.6) to an OD_600_ of 0.75. The mixtures of transcription factors (1 ml) and promoters (100 μl) were infiltrated into tobacco leaves by needleless syringes. *Nicotiana benthamiana* plants were grown in a growth chamber with a light/dark cycle of 16:8 h, at 24 °C. Four-week-old plants were prepared for injection. Enzyme activities of firefly luciferase and renilla luciferase were assayed using dual-luciferase assay reagents (Promega), at 3 d after infiltration. For each transcription factor–promoter interaction, at least three independent experiments were performed, with four replicates in each experiment.

### One-hybrid screening

Y1H assay was carried out using the Matchmatch™ Gold Yeast One-Hybrid System (Clontech), as described in the manufacturer’s protocol. The *CitTPS16* transcriptional promoter was cloned into pAbAi to construct the pAbAi-bait and the full-length *CitERF71* was subcloned into pGADT7 to construct the AD-prey vector (primers are listed in Supplementary Table S1). The pAbAi-baits were transformed into Y1HGold and were screened on selective synthetic dextrose medium (SD) uracil. Colony PCR analysis (Matchmaker Insert Check PCR Mix 1; Clontech) was used to confirm that the plasmids had integrated correctly into the genome of Y1HGold. After determining the minimal inhibitory concentration of aureobasidin A (AbA) for the bait strains, the AD-prey vectors were transformed into the bait strain and screened on an SD/-Leu/AbA plate. All transformations and screenings were performed three times. Autoactivation and transcription factor–protein interaction analysis were conducted according to the manufacturer’s protocol.

### EMSA

To express the recombinant CitERF71 protein, the coding sequence of *CitERF71* was cloned into the pET6×HN vector (Clontech) using the primers listed in Supplementary Table S1, and then expressed in *E. coli* Rosetta BL21 (DE3) pLysS (Promega). Expression and purification of the recombinant protein were performed as described for the TPS enzyme. The EMSA was conducted using the LightShift^®^ Chemiluminescent EMSA kit (ThermoFisher Scientific), according to the manufacturer’s instructions. The *CitTPS16* promoter fragments contain two GCC-box-like sequences (Supplementary Table S2). The double-stranded probes were made by annealing separately synthesized strands, with 3' biotin labeling. Binding assay was carried out in accordance with the published protocol (Nieuwenhuizen *et al.*, 2015).

### Statistical analysis

The two-sample significance test was calculated using single-factor ANOVA (Microsoft Excel, 2013 version). The least significant difference (LSD) at the 5% level was calculated for multiple groups using DPS7.05 (Zhejiang University, Hangzhou, China).

## Results

### Recombinant CitTPS16 protein catalyzes *in vitro* formation of *E*-geraniol

**Fig. 1.**
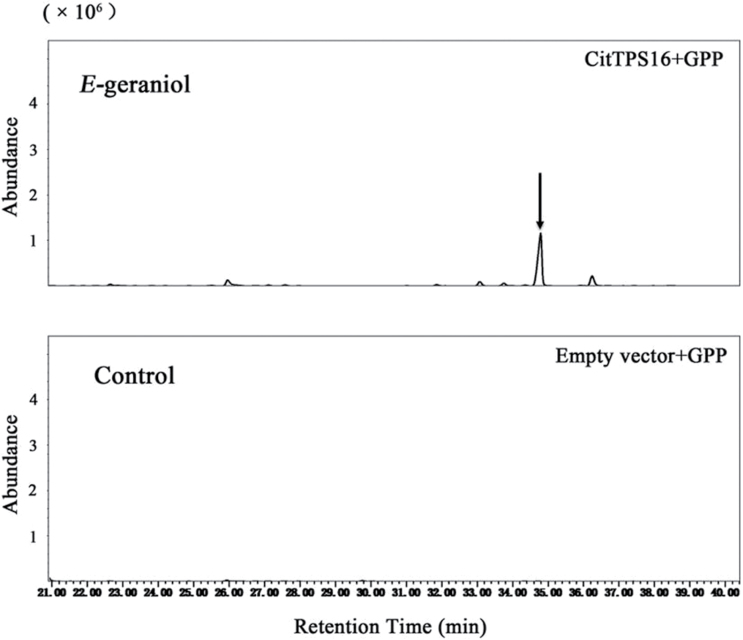
Identification of enzymatic products after incubating recombinant CitTPS16 proteins with geranyl diphosphate. The recombination enzyme expressed in *E*. *coli* was purified by Ni^2+^ affinity and gel filtration chromatography. After solvent extraction, volatile terpenes were analyzed by enantioselective GC-MS.

**Fig. 2.**
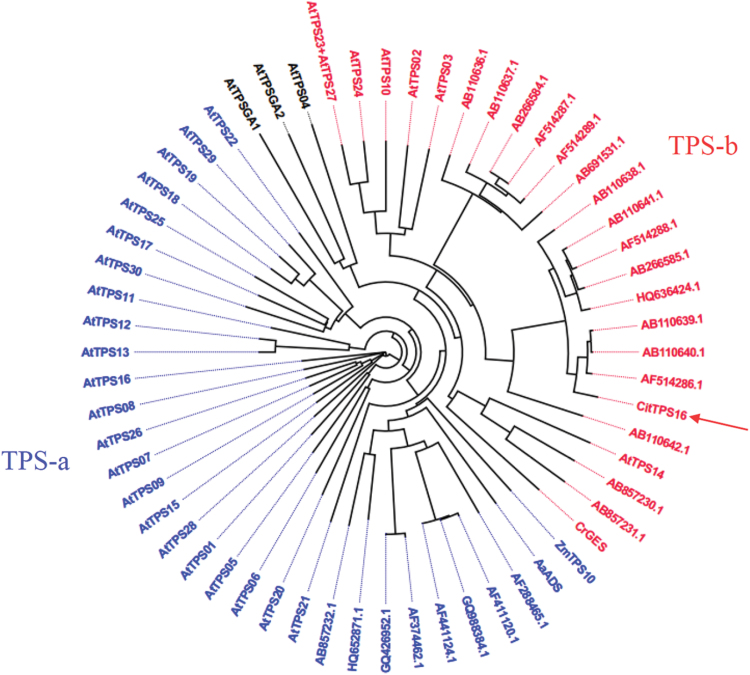
Phylogenetic analysis of CitTPS16 from sweet orange and Arabidopsis. The amino acid sequences were obtained from The Arabidopsis Information Resource or the National Center for Biotechnology Information database. The amino acid sequences were analyzed with ClustalX (v. 1.81). (This figure is available in colour at *JXB* online.)

To characterize genes that are associated with formation of *E*-geraniol, members of the *TPS* family were cloned and expressed in *E. coli.* Enzymatic activity assay showed that multiple volatile terpenoids were produced from 10 recombinant sweet orange TPS proteins (Table 1). One member of the TPS family, CitTPS16 (Supplementary Fig. S1), was able to catalyze *in vitro* formation of *E*-geraniol using GPP as substrate (Fig. 1), and had no activity toward FPP. The most abundant transcripts of *CitTPS16* were detected in fruit tissue when compared with flower, leaf, and callus of sweet orange (http://citrus.hzau.edu.cn/cgi-bin/orange/gene/Cs7g17670.1). CitTPS16 encodes a 292 amino acid protein, belonging to the TPS-b subfamily based on phylogenetic analysis (Fig. 2). Members of the Arabidopsis TPS-b subfamily function as monoterpene synthases *in planta*, using GPP as substrate in the presence of divalent Mg^2+^ cations (Aubourg *et al.*, 2002). CitTPS16 is clustered with plant TPSs that are associated with *E*-geraniol formation, including rough lemon RlemTPS3 and rose CrGES, sharing 63% and 55% similarity at the amino acid level, respectively.

**Table 1.** Enzyme activity assay of recombinant Newhall sweet orange TPS proteins

Protein	Gene ID	Substrate	Product 1 (%)	Product 2 (%)	Product 3 (%)	Product 4 (%)
TPS04	Cs3g04360.1	GPP	Limonene	Others		
			100.00	0.00		
TPS16	Cs7g17670.1	GPP	*E*-Geraniol	Linalool	Limonene	Others
			87.07	7.11	5.81	0.00
TPS21	Cs8g14120.1	GPP	β-Ocimene	*E*-Ocimene	3-Carene	Others
			74.81	5.72	2.38	17.09
TPS03	Cs5g23510.1	FPP	Bicyclosesquiphellandrene	β-Elemene	(–)-α-Copaene	Others
			48.41	10.81	10.24	30.54
TPS07	Cs4g12120.2	FPP	α-Cubebene	(+)-δ-Cadinene	Others	
			44.85	26.43	47.39	
TPS11	Cs4g12460.1	FPP	α-Bergamotene	β-Bisabolene	Sesquiphellandrene	Others
			62.17	15.41	6.95	15.48
TPS24	orange1.1t02008.1	FPP	δ-Cadinene	(–)-β-Elemene	Nerolidol	Others
			35.26	34.74	30.00	0.00
TPS25	Cs4g12350.1	FPP	(+)-β-Selinene	(–)-α-Selinene	(–)-β-Elemene	Others
			34.38	31.56	15.11	18.96
TPS26	Cs4g12400.1	FPP	Bicyclogermacrene	Aromadendrene	Elixene	Others
			44.87	18.95	9.29	26.88
TPS33	Cs4g12090.1	FPP	Germacrene D	Curcumene	Bicyclogermacrene	Others
			33.01	8.91	8.25	49.83

### Transient overexpression of *CitTPS16* in sweet orange increased production of *E*-geraniol

**Fig. 3.**
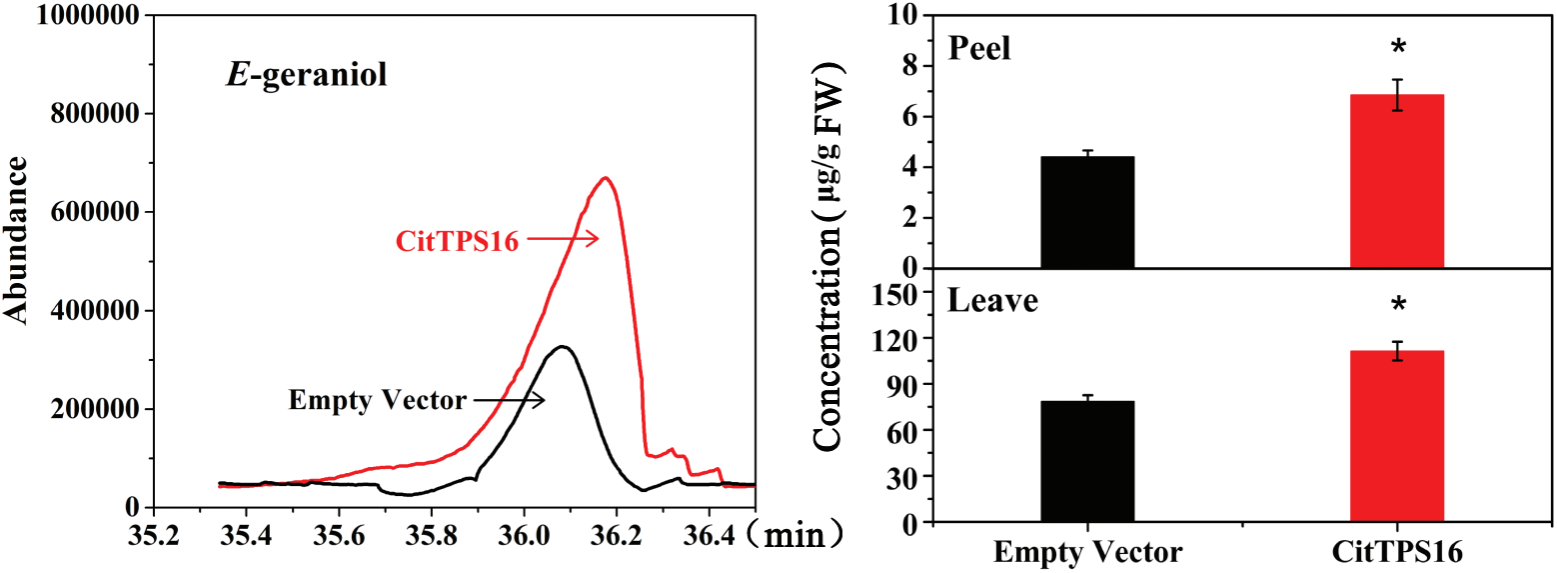
Transient overexpression of *CitTPS16* in Newhall sweet orange. Peels and leaves were infiltrated with *CitERF71* under the control of the *Cauliflower mosaic virus* 35S promoter. After infiltration, the fruit peels and plants were grown for 5 d and 7 d, respectively. The content of *E*-geraniol was detected using GC-MS. Data represent the mean ± SEs of three biological replicates. Asterisks indicate significant differences of values in columns (*P<0.05). (This figure is available in colour at *JXB* online.)

A rapid and efficient transient overexpression assay was chosen to examine the function of *CitTPS16 in vivo*, due to the difficulty of stable transformation systems in perennial fruit. Efficient transient expression systems have been used in orange (Yin *et al.*, 2016) and persimmon (Min *et al.*, 2012). To understand further the role of *CitTPS16* in biosynthesis of *E*-geraniol, homologous transient overexpression was carried out in both fruits and leaves of Newhall sweet orange. The *E*-geraniol content of the peels and leaves infiltrated with *CitTPS16* was 6.85 μg g^–1^ and 111.32 μg g^–1^, respectively, representing a significant (*P*<0.05) increase compared with peel (4.42 μg g^–1^) and leaves (78.50 μg g^–1^) infiltrated with empty vector. These results indicated that introducing *CitTPS16* in Newhall sweet orange accelerated *E*-geraniol biosynthesis (Fig. 3).

### Expression profile of *CitERF71* correlated with *CitTPS16* in sweet orange fruit

**Fig. 4.**
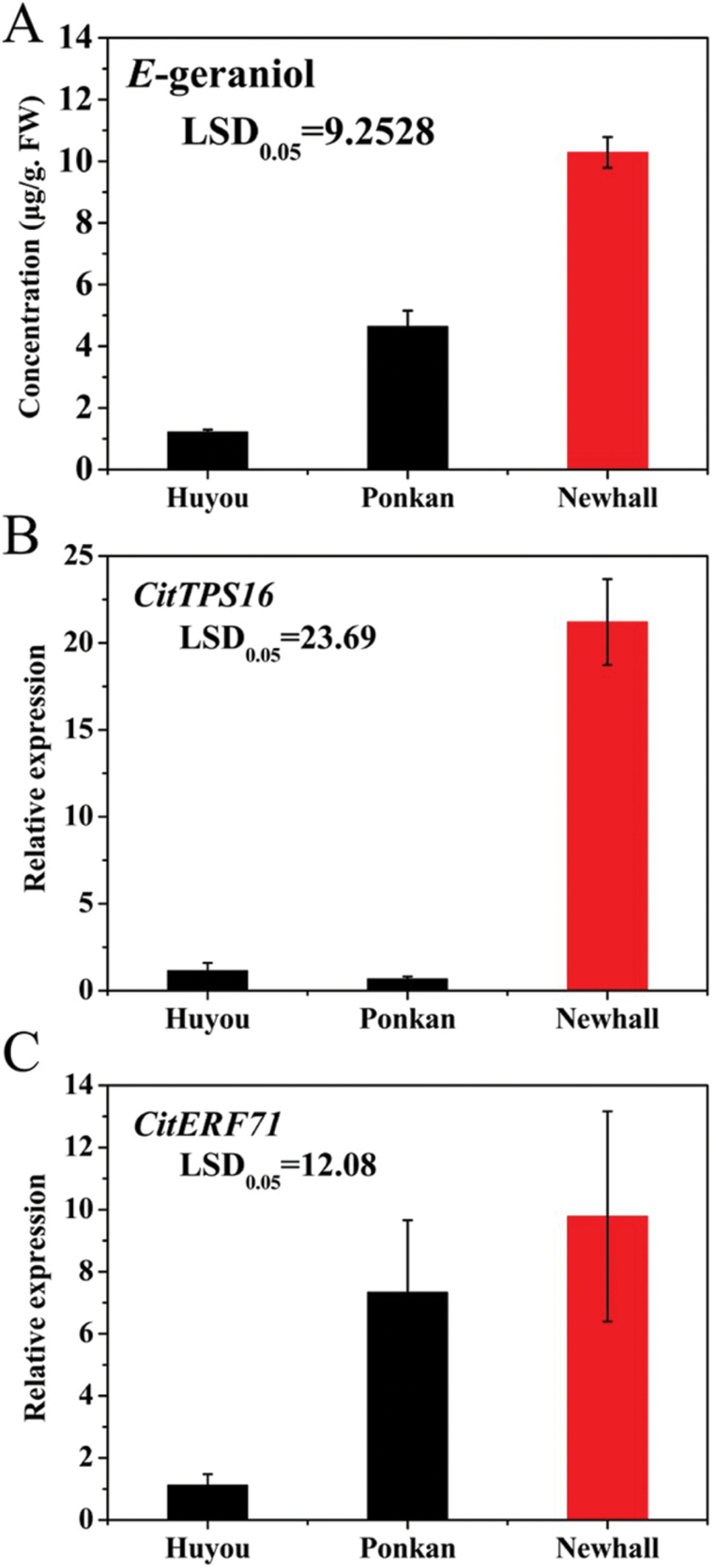
Content of *E*-geraniol and gene expression in citrus fruit. (A) Content of E-geraniol produced by the hybrid Huyou (*C. paradisi*), mandarin Ponkan (*C. reticulata* Blanco), and Newhall sweet orange (*C. sinensis* Osbeck) fruit. (B) Expression profile of *CitTPS16*. (C) Expression profile of *CitERF71*. Data represent the mean ± SEs of three biological replicates. (This figure is available in colour at *JXB* online.)

AP2/ERFs have been reported to be involved in production of volatile terpenoids in plants (Yu *et al.*, 2012; Li *et al.*, 2015; Shen *et al.*, 2016). To investigate regulation of *CitTPS16* expression and *E*-geraniol formation, changes in transcript levels of *CitERF* genes were analyzed in different varieties and during fruit development. The highest content of *E*-geraniol was detected in Newhall sweet orange fruit, while hybrid Huyou and mandarin Ponkan fruit exhibited low levels (Fig. 4). Correspondingly, the most abundant transcript levels of *CitTPS16* were observed in Newhall (Fig. 4). The transcription factor gene *CitERF71* also exhibited the highest transcript levels in Newhall sweet orange, similar to that of *CitTPS16*.

**Fig. 5.**
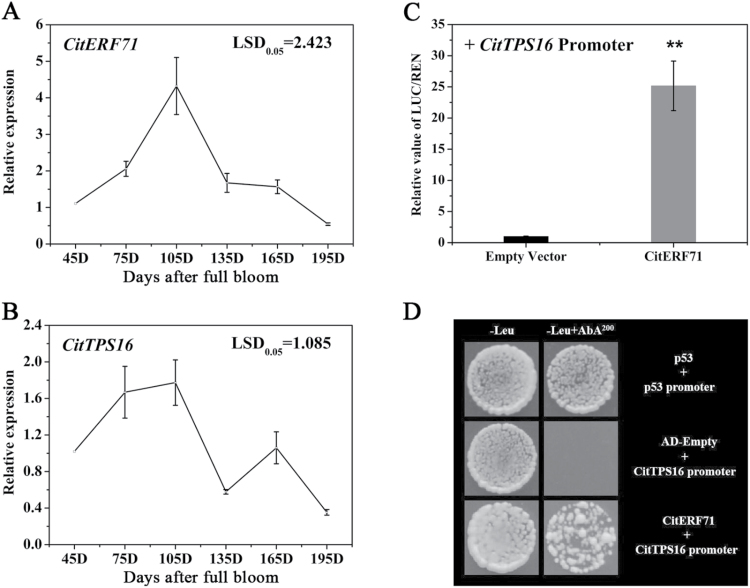
Expression and regulation of genes related to production of *E*-geraniol. (A) Changes in expression of *CitERF71* during Newhall sweet orange ripening. (B) Changes in expression of *CitTPS16* during Newhall sweet orange ripening. Gene expression was calculated relative to the fruit 45 d after full bloom (set as 1). Data represent the mean ±SEs of three biological replicates. The values of LSD represent the significant differences at *P*<0.05. (C) *In vivo* interaction between CitERF71 and the target promoter of *CitTPS16.* Samples were infiltrated into *N. benthamiana* leaves. Firefly luciferase (LUC) and renilla (REN) luciferase were assayed 3 d after infiltration. The ratio of LUC/REN to the empty vector plus promoter was used as a calibrator (set as 1). Data represent the mean ± SEs of four biological replicates. Asterisks indicate significant differences (**P<0.01). (D) Yeast one-hybrid analysis of CitERF71 binding to the *CitTPS16* promoter. Interaction was determined on SD medium lacking leucine in the presence of AbA (–Leu+AbA^200^). AD-p53 and pAbAi-p53 were used as positive controls. AD-empty and pAbAi-*CitTPS16* were used as negative controls.

To test further the correlation between *CitTPS16* and *CitERF71*, transcript levels of these two genes were analyzed during Newhall sweet orange ripening. As shown in Fig. 5A and B, expression of *CitTPS16* and *CitERF71* increased from 60 DAFB, peaked at ~105 DAFB, and then decreased in ripe fruit at 195 DAFB. Regression analysis showed that transcript levels of *CitTPS16* was positively correlated with that of *CitERF71* (*R*^2^=0.624, *P*<0.05). These results suggested that *CitERF71* was a good candidate for having a role in regulating expression of *CitTPS16* in Newhall sweet orange fruit.

Our previous study revealed 126 members of the AP2/ERF transcription factor family in Newhall sweet orange (*C. sinensis* Osbeck) (Xie *et al.*, 2014). Phylogenetic analysis showed that CitERF71 belongs to the ERF IX a subfamily, clustering with Arabidopsis AtERF1, 2, and 13, *Catharanthus roseus* CrORCA3, and *Nicotiana tabacum* NtERF189 (Supplementary Fig. S3). Transcriptional regulation of secondary metabolites was observed by homologs of *CitERF71*, such as involvement of *CrORCA3* in regulating the pathway of jasmonate-inducible terpenoid (van der Fits and Memelink, 2000). These results prompted us to study a possible role for CitERF71 in transcriptional regulation of *CitTPS16* in sweet orange.

### Promoter activation of *CitTPS16* by CitERF71

To test if CitERF71 could regulate expression of *CitTPS16*, a dual-luciferase assay was performed. As expected, a *trans*-activation effect was observed for CitERF71 on *CitTPS16* expression (Fig. 5C). In order to confirm further the specific activation effect of ERF71 on the *CitTPS16* promoter (base pairs –2473 to +1), the activities of 48 CitERFs with the cloned *CitTPS16* promoter were analyzed (Supplementary Fig. S2). The strongest induction was observed for activation of *CitTPS16* by CitERF71, with ~25-fold induction. A Y1H verified that CitERF71 could directly bind to the *CitTPS16* promoter (Fig. 5D). These results indicated that CitERF71 could interact with and activate the *E*-geraniol biosynthetic gene *CitTPS16*.

### Roles of the motif in CitERF71 and *CitTPS16* promoter interaction

**Fig. 6.**
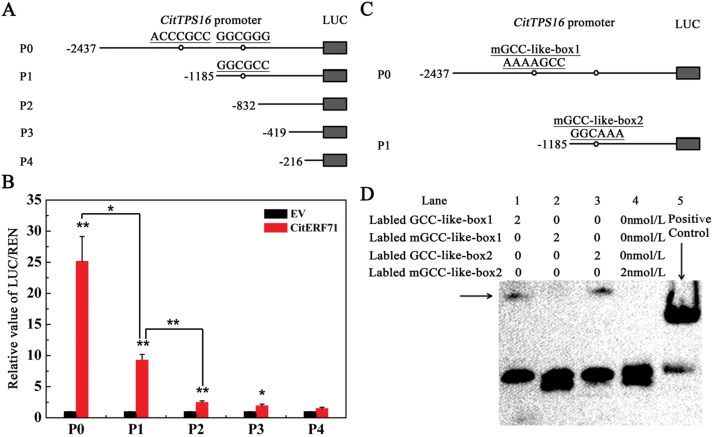
*CitTPS16* promoter deletions and EMSAs. (A) Four deletions were designed to remove two AP2/ERF-related motifs. Schematic representations of promoters (P0–P4) are indicated with lines (promoter length). Circles indicate the presence of GCC-like-boxes. (B) The *in vivo* interactions between CitERF71 and the *CitTPS16* promoter was tested with transient assays in *N. benthamiana* leaves. EV represents empty vector (set as 1). Data represent the mean ± SEs of four biological replicates. Asterisks indicate significant differences (*P<0.05; **P<0.01) (C) Mutant GCC-like-box1 and mutant GCC-like-box2 in the DNA probes used for the EMSA. Schematic representations of promoters (P0 and P1) are indicated with lines (promoter length) and circles (mGCC-like-boxes). (D) EMSAs of 3'-boitin-labeled dsDNA probes with the CitERF71-binding proteins. Two 59 bp *CitTPS16* promoter fragments containing the GCC-like-box and mGCC-like-box were labeled and used as probes. Biotin–EBNA (Epstein–Barr nuclear antigen) control DNA and EBNA extract were used as positive controls. The band corresponding to the CitERF71–GCC-like complex is indicated with a solid black arrow. Lane 1, probe with GCC-like-box1; lane 2, probe with mGCC-like-box1; lane 3, probe with GCC-like-box2; lane 4, probe with mGCC-like-box1; lane 5, positive control (GCC-like-box: GCC-box-like sequence). (This figure is available in colour at *JXB* online.)

To study the roles of different motifs in the promoter of *CitTPS16*, four deletions were produced (Fig. 6A). A major effect of the deletions on *CitTPS16* promoter activity was observed, where the activity was reduced to 36.85% and 9.85% for P1 (base pairs –1185 to 0) and P2 (base pairs –832 to 0), respectively. Additionally, the P2 deletion (containing base pairs –832 to 0) and P3 deletion (containing base pairs –419 to 0) significantly reduced transcription factor and promoter interaction, but did exhibit a low level of expression. There are two hypothetical GCC-box-like motifs [GCC-like-box1, ACCCGCC (base pairs –1499 to–1493); GCC-like-box2, GGCGGG [base pairs –948 to –943)] 5' of the region contained in P2 and none in the P3 or P4 promoter region; some unknown motif within the P3 promoter fragment must be responsible for the low but significant activity observed in P3 (Fig. 6B).

### *In vitro* binding of CitERF71 to the GCC-box-like sequence in the *CitTPS16* promoter

To confirm the binding results, we conducted an EMSA using the CitERF71 recombinant protein together with two 59 bp promoter fragments of *CitTPS16* containing the GCC-like-boxes. GCC-like-box1 and 2 of the *CitTPS16* promoter were both recognized by CitERF71. However, the binding activity disappeared when the GCC-like-box1 core ACCCGCC was changed to AAAAGCC (mGCC-like-box1), and the GCC-like-box2 core GGCGGG was changed to GGCAAA (mGCC-like-box2), respectively (Fig. 6C). These results demonstrated the specificity of binding CitERF71 to the GCC-like-boxes of the *CitTPS16* promoter *in vitro* (Fig. 6D).

## Discussion

Terpenoids are one of the largest groups of secondary metabolites in plants. Generally, biosynthesis of terpenoids is catalyzed by TPS enzymes via either the MVA or the MEP pathway (Sun *et al.*, 2016). Despite the importance of *E*-geraniol to flavor in sweet orange fruits, *E*-geraniol synthase genes have not been identified. Here, we identified the gene encoding a TPS responsible for *E*-geraniol synthesis as well as a transcription factor that is capable of its transcriptional activation. Since citrus is a perennial tree that is difficult to transform stably and takes 4–6 years to obtain transgenic fruits, transient overexpression experiments were conducted to test the biological function of CitTPS16 in *E*-geraniol production. The current results demonstrate that CitTPS16 is associated with biosynthesis of *E*-geraniol based on evidence from both *in vitro* assay of recombinant protein and *in vivo* transient expression in sweet orange.

Functional characterization of the geraniol synthase CrGES from *C. roseus* (Simkin *et al.*, 2013) and RlemTPS3 from rough lemon (Shishido *et al.*, 2012) has been reported. Although CitTPS16 has low sequence similarity to these enzymes (55–63%), it has an identical function in geraniol biosynthesis. Although genes responsible for biosynthesis of *E*-geraniol have been characterized in plants, transcription factors that regulate their expression need to be identified.

Formation of terpenoids can be regulated by transcription factors, including AP2/ERF. In *C. roseus*, CrORCA3, an AP2/ERF transcription factor, was shown to be involved in the regulation of terpenoid synthesis. Overexpression of *CrORCA3* results in accumulation of terpenoid indole alkaloids in *C. roseus* cultured cells (van der Fits and Memelink, 2000). In Newhall sweet orange fruit, *CitAP2.10* regulates (+)-valencene biosynthesis via induction of *CsTPS1* (Shen *et al.*, 2016), while AaERF1 and AaERF2 could bind to the promoter of the artemisinin synthesis gene *AaADS* directly (Yu *et al.*, 2012). In *Zea mays*, ZmERE58 was able to bind directly to the promoter of the *E*-β-farnesene and *E*-α-bergamotene synthesis gene *ZmTPS10* (Li *et al.*, 2015). However, involvement of *AP2/ERF* in transcriptional regulation of *E*-geraniol biosynthesis genes was not characterized. The present study showed CitERF71 binding directly to the promoter of *CitTPS16*, which is responsible for *E*-geraniol formation. Moreover, the correlation between gene expression of the transcription factor and the TPS gene, together with the dual-luciferase assay, Y1H assay, and EMSA, all support the conclusion that CitERF71 is involved in transcriptional regulation of the *CitTPS16* gene. However, a direct effect of CitERF71 on *E*-geraniol biosynthesis requires further investigation.

It has been reported that CitERF71-related genes bind to GCC-box-like sequences and act as activators in plants (Shoji and Hashimoto, 2012). The binding capacities of five group IXa ERFs (tobacco ERF189, tobacco ERF163, *Catharanthus* ORCA3, Arabidopsis AtERF13, and Arabidopsis AtERF1) to the ERF189 recognition site in the promoter of the tobacco putrescine *N*-methyltransferase gene were examined *in vitro*, as well as transactivation of the promoter in a tobacco transient expression assay. The more similar the protein sequences were to ERF189, the more effective these ERFs were in these functional assays. Eight previously identified ERF189-binding sites (Shoji *et al.*, 2010; Shoji and Hashimoto, 2011, 2012) conform to an improved consensus sequence, 5'-(A/C)GC(A/C)NNCC(A/T)-3' in which the four underlined bases are absolutely required for ERF189 recognition.

The dual-luciferase and Y1H assays showed that CitERF71 directly binds to and *trans*-activates the *CitTPS16* promoter, containing the 5'-(A/C)GC(A/C)NNCC-3' sequence. However, our results indicated that CitERF71 could not bind to a 59 bp fragment with the 5'-AGCAAGCC-3' motif. Our EMSA results indicated that 5'-ACCCGCC-3' and 5'-GGCGGG-3' and not 5'-AGCAAGCC-3' were the core motifs affecting the interaction.

It has been reported that the transcription factors ERF189, ORCA3, and AtERF13 contain a central DNA-binding domain (Supplementary Fig. S4), an N-terminal acidic domain rich in serine, glutamate, and aspartate, and a short serine-rich stretch at the C-terminal region (van der Fits and Memelink, 2001; Shoji *et al.*, 2010). The N-terminal acidic domain in ERF189 was shown to be required for activation of its target promoter, in accordance with the acidic activation domain found in ORCA3. The N-terminal acidic region in the IXa subfamily ERFs thus appears to act as the activation domain of transcription factors. A cluster of serine-rich residues found in several members of the AP2/ERF-domain family has been reported to be inhibitory in the strictosidine synthase promoter in *C. roseus* cells (van der Fits and Memelink, 2001). In contrast, no inhibitory effect of the serine-rich stretches in ORCA3, ERF189, and ERF163 was reported, when analyzed in the *PMT2* promoter in tobacco cells. These observations imply that this discrepancy might be due to the differences in the promoters and the transient expression systems used (Shoji and Hashimoto, 2012). Moreover, N-terminal acidic domain analysis suggested that CitERF71 is likely to be an activator in plant secondary metabolism in *Citrus*.

In conclusion, we have identified a terpene synthase, CitTPS16, that synthesizes the important flavor volatile *E*-geraniol. The transcription factor CitERF71 directly binds to ACCCGCC and GGCGGG motifs in the *CitTPS16* promoter, activates the promoter, and therefore probably has a function in transcriptionally regulating *E*-geraniol production in Newhall sweet orange fruit.

## Supplementary data

Supplementary data are available at *JXB* online.

Table S1. Primers used in the present study.

Table S2. Complementary 59 bp oligonucleotides for EMSA.

Fig. S1. SDS–PAGE analysis of recombinant *CitTPS16* protein.

Fig. S2. *In vivo* interaction between 48 CitERFs and the *CitTPS16* promoter.

Fig. S3. Phylogenetic analysis of plant AP2/ERF transcription factors.

Fig. S4. Sequence alignment of AP2/ERF transcription factors.

## Supplementary Material

Supplementary_Tables_Figures
