# Supplementary material for: Transcription factor CitERF71 activates the terpene synthase gene CitTPS16 involved in the synthesis of E-geraniol in sweet orange fruit
Source: J Exp Bot. 2017 Sep 7;68(17):4929–38. doi: 10.1093/jxb/erx316 (PMC5853461; doi:10.1093/jxb/erx316)
Supplement: Supplementary_Tables_Figures [file erx316_suppl_supplementary_tables_figures.pdf]

Transcription factor CitERF71 activates terpene synthase CitTPS16 that is associated with the synthesis of E-geraniol in sweet orange fruit

Xiang Li, Yaying Xu, Shuling Shen, Xueren Yin, Harry Klee, Bo Zhang†, Kunsong Chen

†Correspondence: bozhang@zju.edu.cn

Supplementary files:

Table S1. Primers used in the present study.

Table S2. Complementary 59 bp oligonucleotides for EMSA.

Fig. S1. SDS-PAGE analysis of recombinant *CitTPS16* protein.

Fig. S2. *In vivo* interaction between 48 CitERFs and *CitTPS16* promoter.

Fig. S3. Phylogenetic analysis of plant AP2/ERF transcription factors.

Fig. S4. Sequence alignment of AP2/ERF transcription factors.

Table S1. Primers used in the present study.

**Primers**	**Sequence(5'-3')**	**Description**
CitTPS16F	GGGTGGCCAAGCATGATATT	RT-QPCR of *CitTPS16*
CitTPS16R	GATAGGGTTCATGTCTGGTCC	RT-QPCR of *CitTPS16*
CitERF71F	ATGTTGAGCCCCCGGTTAG	RT-QPCR of *CitERF71*
CitERF71R	GTCTAAAGGACACAACGGATGAT	RT-QPCR of *CitERF71*
Citrus actin F	CATCCCTCAGCACCTTCC	RT-QPCR of Citrus actin
Citrus actin R	CCTCTTCACGATTCCAACC	RT-QPCR of Citrus actin
pET-CitTPS16F	**CTGCAG**GTAGGCGATCTGCCGATTACGGG	Subclone to pET Vector of *CitTPS16*
pET-CitTPS16R	**GAATTC**GCCTCTTAGTGTATTGCTTGCAC	Subclone to pET Vector of *CitTPS16*
SK-CitTPS16F	CGCGGTG**GCGGCCGC**ATGGCTCTTAATCTGCT	Subclone to pGreen-SK Vector of *CitTPS16*
SK-CitTPS16R	GGGGGATCC**ACTAGT**TTAGTGTATTGCTTGCAC	Subclone to pGreen-SK Vector of *CitTPS16*
pET-CitERF71F	ATCACAACG**CTGCAG**GTATGAACAGAGAAAATATG	Subclone to pET Vector of *CitERF71*
pET-CitERF71R	CGGCCGCCA**GAATTC**GCTCACCCGGGTCTAAAGG	Subclone to pET Vector of *CitERF71*
SK-CitERF71F	ATGAACAGAGAAAATATGTCCGA	Subclone to pGreen-SK Vector of *CitERF71*
SK-CitERF71R	ACAGGAAATCTGGGCCCACT	Subclone to pGreen-SK Vector of *CitERF71*
AD-CitERF71F	GAGGCCAGT**GAATTC**ATGAACAGAGAAAATATGTCCG	Subclone to pGADT7 AD Vector of *CitERF71*
AD-CitERF71R	GAGCTCGAT**GGATCC**TCACCCGGGTCTAAAGGACA	Subclone to pGADT7 AD Vector of *CitERF71*
pAbAi-CitTPS16F	CTTGAATTC**GAGCTC**GGGTATATGACCGCTATATGTGTG	Subclone to pAbAi Vector of *CitTPS16*
pAbAi-CitTPS16R	TGCCTCGAG**GTCGAC**CATGACTTAGAATATAATTTCAAGG	Subclone to pAbAi Vector of *CitTPS16*
LUC-CitTPS16(P0)F	TTCTAGA**GCGGCCGC**GGGTATATGACCGCTATATGTGTG	Subclone to pGreen-LUC Vector of *CitTPS16*
LUC-CitTPS16(P1)F	TTCTAGA**GCGGCCGC**CCAATATACCAATTCGGTTCAGT	Subclone to pGreen-LUC Vector of *CitTPS16*
LUC-CitTPS16(P2)F	TTCTAGA**GCGGCCGC**CGTGTCAAGCTCTCATAGAGAGA	Subclone to pGreen-LUC Vector of *CitTPS16*

LUC-CitTPS16(P3)F	TTCTAGA**GCGGCCGC**CACACATGTCATCACATCAT	Subclone to pGreen-LUC Vector of *CitTPS16*
LUC-CitTPS16(P4)F	TTCTAGA**GCGGCCGC**GTCAATGAGTCACATGTCATG	Subclone to pGreen-LUC Vector of *CitTPS16*
LUC-CitTPS16R	TGGCGTCTT**CCATGG**CTTAGAATATAATTTCAAGG	Subclone to pGreen-LUC Vector of *CitTPS16*

The restriction enzyme sites are labeled.

Table S2. Complementary 59 bp oligonucleotides for EMSA.

**Name**	**Sequence (5'-3')**
GCC-box-like sequence 1	GAATGGGCGATAGAGCTCATACTT**ACCCGCC**TCTCTTCACATTCAAACTTCTAATATTA
mGCC-box-like sequence 1	GAATGGGCGATAGAGCTCATACTT**AAAAGCC**TCTCTTCACATTCAAACTTCTAATATTA
GCC-box-like sequence 2	AATTTACAAAATTATAGAAGTCAAATTACGACGTCGTAAATCT**GGCGGG**ACTTTAATGG
mGCC-box-like sequence 2	AATTTACAAAATTATAGAAGTCAAATTACGACGTCGTAAATCT**GGCAAA**ACTTTAATGG

The GCC-like-box motif and mGCC-like-box motif are labeled.

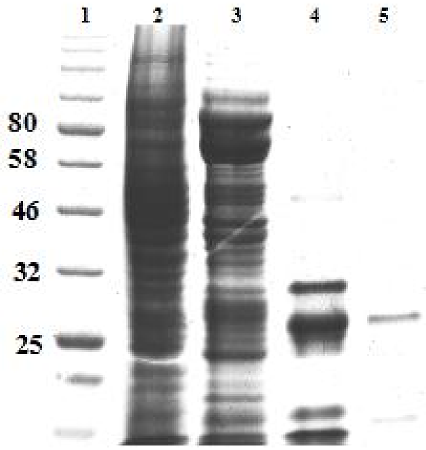

Fig. S1 SDS-PAGE analysis of recombinant CitTPS16 protein. M: molecular weight markers ranging from 10 to 180 kDa, (1) Marker, (2~5) recombinant CitTPS16 proteins.

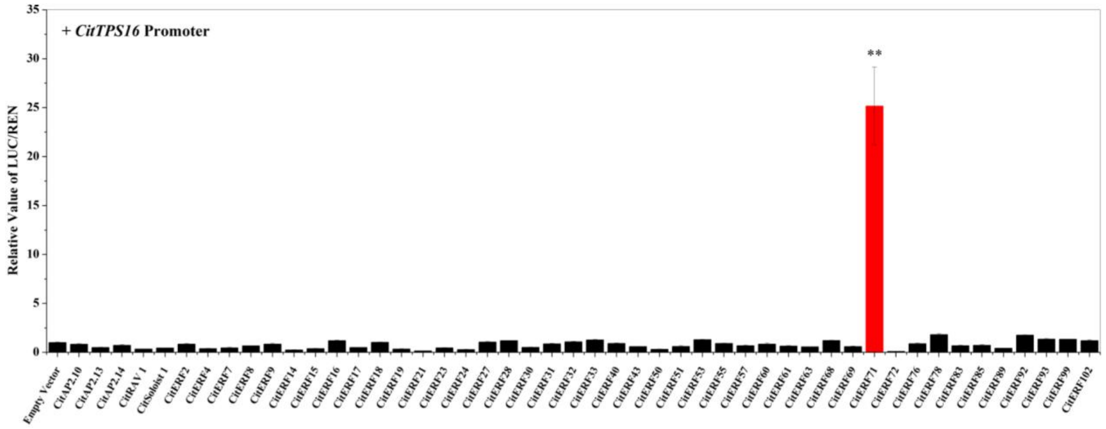

Fig. S2 *In vivo* interaction between ethylene-responsive factors and *CitTPS16* promoter. Associations of TFs and promoter were obtained from transient assays in *N. benthamiana* leaves. For the transcription factor-promoter interactions, three independent experiments were performed (four replicates in each experiment). Firefly luciferase (LUC) and renilla (REN) luciferase were assayed 3d after infiltration. The ratio of LUC/REN to the empty vector plus promoter was used as a calibrator (set as 1). Error bars indicate SE from four biological replicates (***P*<0.01).

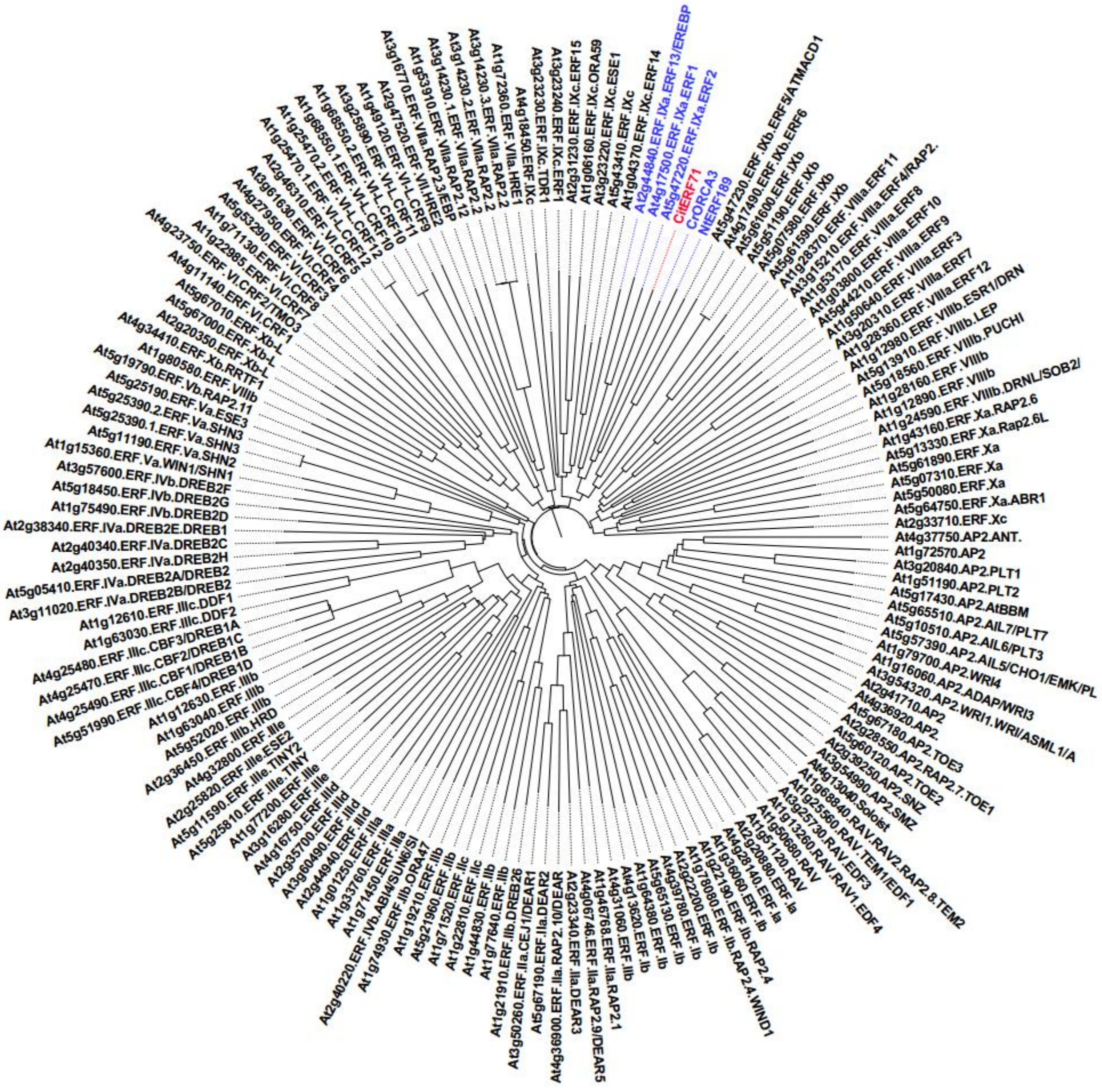

Fig. S3. Phylogenetic analysis of plant AP2/ERF genes. CitERF71 was donated as red font. The amino acid sequences were obtained from The Arabidopsis Information Resource or the National Center for Biotechnology Information database. The amino acid sequences were analyzed with ClustalX (v. 1.81).

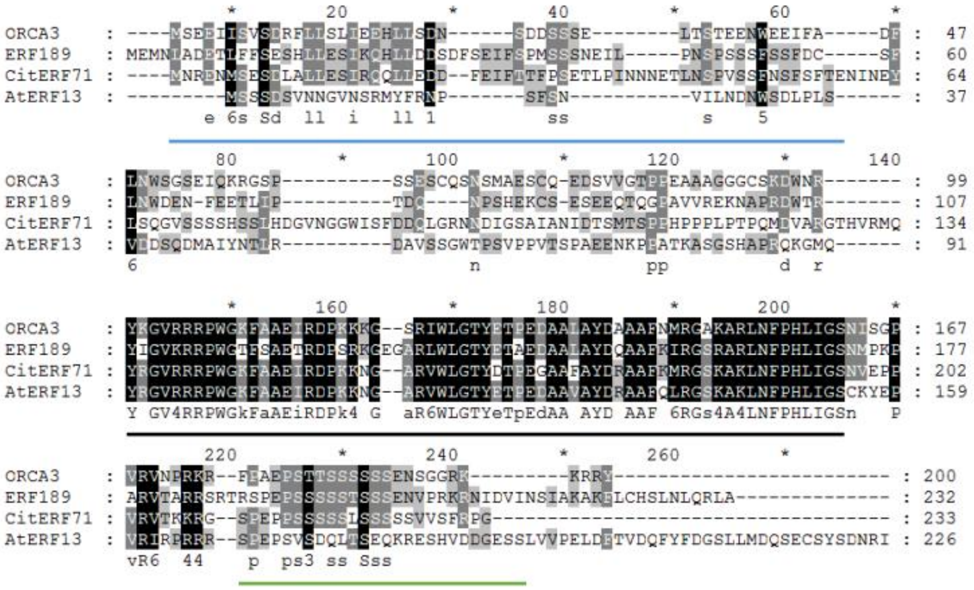

Fig. S4 Sequence alignment of AP2/ERF transcription factors. Alignment of protein sequences of *N. tabacum* ERF189, *C. roseus* ORCA3, Arabidopsis AtERF13 and Newhall sweet orange CitERF71. Residues identical in at lease two sequences are shaded in black, and dashes indicate gaps introduced to maximize the alignment. The AP2/ERF DNA-binding domain is underlined (black), the N-terminal Ser/Asp/Glu-rich acidic domain (blue), and the short Ser-stretch (green).
